# Would you want to know? Public attitudes on early diagnostic testing for Alzheimer's disease

**DOI:** 10.1186/alzrt206

**Published:** 2013-09-06

**Authors:** Elizabeth M Wikler, Robert J Blendon, John M Benson

**Affiliations:** 1 Harvard Graduate School of Arts and Sciences, 14 Story Street, 4th Floor, Cambridge, MA 02138 USA; 2 Department of Health Policy and Management, 677 Huntington Avenue, Kresge Building, Room 402, Boston, MA 02115 USA

**Keywords:** Alzheimer's disease, medical testing, predictive testing, medical decision-making, public attitudes, preclinical

## Abstract

**Introduction:**

Research is underway to develop an early medical test for Alzheimer's disease (AD).

**Methods:**

To evaluate potential demand for such a test, we conducted a cross-sectional telephone survey of 2,678 randomly selected adults across the United States and four European countries.

**Results:**

Most surveyed adults (67%) reported that they are "somewhat" or "very likely" to get an early medical test if one becomes available in the future. Interest was higher among those worried about developing AD, those with an immediate blood relative with AD, and those who have served as caregivers for AD patients. Older respondents and those living in Spain and Poland also exhibited greater interest in testing. Knowing AD is a fatal condition did not influence demand for testing, except among those with an immediate blood relative with the disease.

**Conclusions:**

Potential demand for early medical testing for AD could be high. A predictive test could not only advance medical research, it could transform political and legal landscapes by creating a large constituency of asymptomatic, diagnosed adults.

## Introduction

In 2011, an international team of experts revised the diagnostic criteria and guidelines used to identify Alzheimer's disease (AD). In recognition of recent scientific discoveries, the group proposed a research agenda focused on early detection of AD, particularly when the disease is in a preclinical stage: after key biological changes have started to occur in the brain, but before the onset of noticeable symptoms [1]. Their hope was that identifying the disease in this preclinical stage will facilitate the development of new treatments to slow or halt the progression of the disease [2]. Across the globe, research on the predictive value of early medical tests used to detect Alzheimer's disease during this preclinical phase is underway, and is showing promising results [3,4].

Although nascent, efforts to create an early medical test for Alzheimer's disease using both genetic information and disease biomarkers are gaining traction and, in the future, may be available for broad populations of asymptomatic patients outside of the research environment. While this could result in tremendous breakthroughs regarding treatment technologies, it raises practical, ethical and financial questions for individuals and communities across the globe. Most of all, people all over the world will face a decision: should they get tested? Would they want to know whether they will get this fatal, untreatable disease?

This paper draws on public opinion data from four Western European countries and the United States to assess potential international demand for early diagnostic testing for Alzheimer's disease. We also explore some of the factors associated with high and low levels of interest in early medical testing for AD, employing constructs from the Health Belief Model, a commonly used theoretical model that predicts utilization of health services [5]. Our results suggest that demand for Alzheimer's testing among asymptomatic patients could be high across all five countries, particularly among those who perceive themselves to be at high risk for the disease.

### Early medical testing for AD

For decades, scientists have been working to develop a reliable, predictive test for Alzheimer's disease. In 1994, genetic testing for late-onset Alzheimer's disease became available primarily in research settings, offering patients a probabilistic measure of their risk for the disease by analyzing their apolipoprotein E (ApoE) genotype. Individuals with two ApoE ε4 alleles have more than seven times increased risk of developing AD than those with the ApoE ε3 allele [6]. However, the ApoE ε4 allele is neither a necessary nor sufficient predictor of the disease, and the association between ApoE ε4 allele and AD has been shown to vary by race and ethnicity [7]. For these reasons and others - including the test's low sensitivity and specificity, the difficulty of interpreting probabilistic results, and the lack of prevention options - experts have largely opposed widespread clinical adoption of this genetic test [8].

Other initiatives have focused on disease biomarkers - particularly those measuring changes in amyloid beta accumulation, synaptic and neuronal function, and brain structure - in hopes of developing tests that can track pathophysiological changes related to the development of the disease [9-13]. If these new tests can more accurately predict the onset of Alzheimer's disease, it is feasible that at some point in the future, they could be incorporated into a broader set of tests used for the early detection and diagnosis of Alzheimer's disease and made available to patients outside research settings.

### Health belief model

In this paper, we drew on the Health Belief Model (HBM) to develop our hypotheses about the social and demographic factors that predict interest in AD testing. The HBM is a theoretical framework developed by the U.S. Public Health Service to help explain low participation rates in disease prevention programs [5,14,15]. The core premise of the model is that health behaviors, such as testing, are driven by personal beliefs about health conditions, for example, the extent to which a person feels threatened by a particular health condition, and the costs and benefits of the strategies available for its detection and treatment [14]. Individuals assess threat based on their perceptions of how susceptible they are to an illness and the severity of the illness, including its impact on their future [16]. In this study, we expected that measures of perceived risk would positively predict interest in testing, whereas measures of disease severity, including knowing the disease was fatal, would inversely predict interest [17-19]. Previous work also suggests that perceived benefits, such as enhanced planning and decision-making abilities around future care options, predict interest in testing, whereas perceived costs, such as lack of treatment, depress interest and uptake [20-22].

## Methods

### Survey data and study participants

The data for this paper come from an international telephone survey with a randomly-selected sample of 2,678 adult respondents age 18 and older, drawn from five countries: France, Germany, Poland, Spain and the United States. The Harvard School of Public Health and Alzheimer Europe commissioned the survey to assess public understanding about Alzheimer's disease. The fieldwork was conducted from 7 to 27 February 2011 by TNS which is one of the largest, independent research companies in the world and is based in London with branches in each of the five countries surveyed. In each of the five countries, interviews were conducted both by landline telephone using random-digit dialing and by cell phone using numbers chosen randomly from a list of cell phone numbers across the country. Interviews were conducted in the language of each country. In the United States, interviews were conducted in both English and Spanish. The average length of an interview was 12 minutes [23].

The survey, which has been described elsewhere, focused on eight broad topics, ranging from levels of public concern about the disease to public beliefs about whether an effective treatment is available to slow the progression of the disease [23]. In this analysis, we focused on results related to interest in future early diagnostic testing for the disease, should such a test become available.

In December 2010, the Institutional Review Board at the Harvard School of Public Health ruled that this study was not human subject research (Protocol #19950-101).

Informed consent was obtained in the following manner. The interviewer told potential respondents that the call was being made on behalf of the Harvard School of Public Health. They were also told that the information they provided would remain confidential and be used for research purposes only. Potential respondents were not pressured into responding, and could have chosen not to be interviewed. Personal identifiers such as telephone numbers were discarded.

### Statistical analyses

Nonresponse in telephone surveys produces some known biases in survey-derived estimates, because participation tends to vary for different subgroups of the population. To compensate for these biases, the sample data were weighted to reflect the actual composition of the adult population in the surveyed countries, calculated on the basis of census data from each country, according to age, gender and region. The sample data were also weighted by telephone status (landline, cell). Other techniques, such a selection within households, were used to help ensure that the sample in each country was representative.

We used chi-squared tests (assessed at the conventional alpha level of 0.05) to evaluate the association between interest in testing and levels of perceived threat, perceived costs and benefits, and social and demographic factors. We then used regression analysis to analyze variables that predicted interest, adjusting for social and demographic factors. Only the final models are shown. As recommended by Strecher and Rosenstock, we did not aggregate items measuring the constructs of the health belief model, despite some measures being moderately interrelated, and instead, evaluated the impact of each measure separately [24]. However, we tested for potential interactions among these variables. As robustness checks, we ran country-specific regression models, and evaluated all models using probit regression.

Our main outcome variable - interest in testing - was derived from the following survey question: "[i]n the future, a medical test might become available that would tell people before they had symptoms whether they will get Alzheimer's disease in the future. If such a test became available, how likely do you think it is that you would get the test - very likely, somewhat likely, not too likely, or not at all likely?" Because studies of genetic testing for other diseases have found that actual take-up rates tend to be lower than rates of expressed interest once tests become available, we focused our analyses on respondents who reported that they are "very likely" to get tested (1, very likely; 0, somewhat/not too/not at all likely) [21]. In additional analyses, we evaluated predictors of being "not at all likely" to get tested, creating a separate dichotomous outcome variable (1, not at all likely; 0, very likely/somewhat/not too). We dropped the "Don't know/Refused" respondents (*n *= 53) from this analysis.

The independent variables in this analysis were drawn from the Health Belief model [5,14]. Measures were broken into categories that evaluate levels of perceived threat and perceived costs and benefits of testing; demographic and psychological variables were also included in the models. Given differences in educational systems across the countries, we divided respondents into three categories: low, middle and high educational attainment. Across all countries, low education captured respondents who completed primary and some secondary schooling; middle education captured respondents who had completed high school and potentially some college; and high education referred to respondents with a college or post-university degree. Race and ethnicity data were collected only in the United States, so models evaluating their impact were conducted only for respondents living in that country. Respondents were broken down into the following categories: white (non-Hispanic), black (non-Hispanic), Hispanic, other (non-Hispanic). The other racial category included respondents who identified as Asian, Pacific Islander, Native American or other.

Reference categories for dichotomous variables were set at zero, and for categorical variables, they were set as the first group. All analyses were conducted using Stata 11 software, which was generated by StataCorp LP, a company located in College Station, Texas in the United States.

## Results

Table 1 shows unadjusted, weighted statistics on the relationship between interest in testing and our independent variables for respondents who expressed they would be "very likely" to take a medical test for AD.

**Table 1:** Characteristics of total sample and subpopulations who are "very likely" to obtain preclinical AD test

		Entire sample		Very likely to get early medical test for AD	
Entire sample				30.0%	[28.1 to 31.9]
Have/had immediate blood relative with AD					
	No	77.1%	[75.2 to 79.0]	27.8%***	[25.7 to 30.1]
	Yes	22.9%	[21.0 to 24.8]	35.3%	[31.2 to 39.7]
Served as decision-maker or caretaker for AD patient					
	No	83.0%	[81.4 to 84.7]	27.9%*	[25.8 to 30.0]
	Yes	17.0%	[15.3 to 18.6]	40.4%	[35.6 to 45.5]
Worried will get Alzheimer's disease					
	Not too/not at all/don't know/refused	55.3%	[53.1 to 57.5]	23.5%**	[21.2 to 26.0]
	Very/somewhat worried	44.7%	[42.5 to 46.9]	38.1%	[35.1 to 41.2]
Health status					
	Fair/poor	20.3%	[18.5 to 22.2]	34%*	[29.8 to 38.4]
	Excellent/very good/good	79.7%	[77.8 to 81.5]	28.9%	[26.8 to 31.1]
Think AD is fatal					
	No/Don't know/refused	55.5%	[53.3 to 57.7]	28.9%	[26.4 to 31.5]
	Yes	44.5%	[42.3 to 46.7]	31.4%	[28.6 to 34.4]
Marital status					
	No	46.3%	[44.1 to 48.6]	28.9%	[26.1 to 31.9]
	Yes	53.7%	[51.4 to 55.9]	30.9%	[28.4 to 33.6]
Expect paid caregiver to be primary caretaker if develop AD					
	No	70.8%	[68.7 to 72.8]	28.6%*	[26.3 to 30.9]
	Yes	29.2%	[27.2 to 31.3]	33.4%	[29.8 to 37.2]
Believe an effective AD treatment is available now or will be in five years					
	No/don't know/refused	32.1%	[30.0 to 34.2]	28.5%	[25.3 to 32.0]
	Yes	67.9%	[65.8 to 70.0]	30.8%	[28.4 to 33.2]
Age					
	18 to 29	23.7%	[21.7 to 25.8]	25.3%**	[21.3 to 29.9]
	30 to 49	37.2%	[35.1 to 39.4]	25.1%	[22.2 to 28.2]
	50 to 64	21.6%	[19.9 to 23.3]	36.9%	[33.1 to 40.9]
	65 to 74	11.6%	[10.2 to 13.0]	38.9%	[33.2 to 45.0]
	75 to 85+	5.9%	[4.9 to 6.9]	34.3%	[27.0 to 42.3]
Gender					
	Male	48.2%	[46.0 to 50.5]	27.7%*	[24.9 to 30.5]
	Female	51.8%	[49.5 to 54.0]	32.2%	[29.6 to 34.9]
Educational attainment					
	Low	32.5%	[30.4 to 34.6]	32.4%*	[29.0 to 35.9]
	Middle	30.2%	[28.1 to 32.3]	32.1%	[28.6 to 35.9]
	High	37.3%	[35.1 to 39.5]	25.9%	[23.0 to 29.0]
Country					
	France	20.8%	[19.0 to 22.6]	26.8%***	[22.9 to 31.2]
	Germany	17.9%	[16.2 to 19.7]	23.6%	[19.7 to 28.0]
	Poland	17.8%	[16.1 to 19.5]	30.5%	[26.2 to 35.2]
	Spain	18.4%	[16.6 to 20.1]	39.6%	[34.9 to 44.4]
	United States	25.1%	[23.1 to 27.0]	29.7%	[26.0 to 33.7]
Race (U.S. Only, *N *= 639)					
	White	68.3%	[64.3 to 72.4]	25.3%	[21.1 to 30.0]
	Black	11.6%	[9.1 to 13.9]	45.1%	[34.6 to 56.0]
	Hispanic	14.1%	[11.3 to 16.9]	34.7%	[25.7 to 44.8]
	Other (Asian, Pacific Islander, Native American, Other)	6.1%	[3.1 to 8.9]	39.1%	[18.1 to 65.2]
Would see doctor if showing symptoms of AD					
	No	9.2%	[7.8 to 10.5]	19%**	[13.3 to 26.6]
	Yes	90.8%	[89.5 to 92.2]	31.4%	[29.4 to 33.5]

*N *= 2,678; *P < .05; **P < .01; ***P < .001 using chi-square tests of differences between expected and observed distributions. Sample sizes vary across categories due to missing data. Confidence intervals in brackets.

### Bivariate analyses

Overall, we found that more than two-thirds of respondents across all countries would be "very likely" (30%) or "somewhat likely" (37%) to obtain an early medical test if it were available in the future; 21.1% would be "not too likely," and 11.9% would be "not at all likely" to pursue testing. Individuals who reported high levels of perceived threat, across a range of measures, were more likely to indicate that they are "very likely" to undergo testing [15,17,25,26]. For example, we found that 35.3% of respondents who had an immediate blood relative with AD report that they were "very likely" to get tested as compared to 27.8% without an affected relative. Similar patterns emerged for those who served as a caretaker or decision-maker (40.4% versus 27.9%), for those who were "very" or "somewhat" worried about getting AD (38.1% versus 23.5%), and for those who were in "fair" or "poor" health (34% versus 28.9%). Unexpectedly, knowing that AD is a fatal condition - a measure of perceived disease severity - had no statistically significant relationship to interest in testing.

Only one of our measures of perceived costs and benefits was associated with interest in testing: those who expected to rely on a paid caregiver if they developed AD, as opposed to a spouse, child, friend or other, were more likely to desire testing (33.4% versus 28.6%).

Our analysis showed that all of the demographic and psychological control variables in our model were statistically significantly related to being "very likely" to pursue testing. Older populations - those aged 50 and above - reported more often than younger populations that they were "very likely" to get an early AD test (for example, 38.9% for 65 to 74 year olds versus 25.3% for 18 to 29 year olds); women were more interested than men (32.2% versus 27.7%); and those with the lowest levels of education were more interested than those with the highest (32.4% versus 25.9%). Country-level differences were also particularly striking - Spain and Poland had the highest shares of respondents reporting that they were "very likely" to pursue testing (39.6% and 30.5%) and Germany the lowest (23.6%).

Lastly, we found that those with potentially stronger coping abilities, as captured by their more active information-seeking style (measured by asking respondents if they would see a doctor if showing symptoms of AD) were more likely to express interest in testing (31.4% versus 19%) [27].

In the United States, whites were less likely than blacks and Hispanics to respond that they would be "very likely" to get an early medical test for AD (25.3% compared to 45.1% and 34.6%, respectively). However, only the difference between whites and blacks was statistically significant.

We tested variables associated with being "not at all likely" to get an early medical test, and found that results largely mirrored those outlined above with one additional finding: those who believed there was a treatment for AD, or that one will become available in the next five years, were less likely to respond that they were "not at all likely" to get tested (10.5% versus 14.1%). This suggested that while treatment optimism may not motivate people to express strong interest in testing, it did prevent people from ruling out testing altogether.

Nevertheless, measures of perceived risk and perceived costs and benefits were interrelated. For example, women and caretakers were more likely to report being worried about getting AD, and having a close blood relative with AD was positively associated with higher levels of treatment optimism. To disentangle these effects, we used multivariate logistic regression to adjust for these correlations and check for potential interactive effects.

### Regression results

Our logistic regression models evaluated interest in testing, adjusting for measures of perceived risk, measures of perceived costs and benefits, and demographic and social controls. Table 2 displays our results. On average, after controlling for social and demographic factors, we find that 28.4% of survey respondents are "very likely" to get a hypothetical, early medical test for AD. Although several factors, including the cost of the test and methods of test administration, may reduce rates of actual take-up once and if an AD test is available, this level of interest is similar to the 25% take-up rates documented among first-degree relatives of AD patients who were part of disease registries and contacted to partake in a randomized clinical trial for a genetic testing for AD [28].

**Table 2:** Logistic Regression results evaluating variables predictive of being "very likely" to obtain preclinical AD test

		Model 1	Model 2
Variables		AOR	AOR
Measures of perceived threat			
	Respondent is or was decision-maker or caretaker for AD patient	1.306*	1.330*
	Worried will get Alzheimer's	1.760***	1.22
	"Excellent/Good/Very Good" Health Status	0.972	0.763
	Worried* Excellent/Good/Very Good Health Status	--	1.610*
	Have/had immediate blood relative with AD	1.312*	0.971
	Think AD is fatal	1.112	0.96
	Immediate blood relative* Think AD is fatal	--	1.783*
Measures of perceived costs and benefits			
	Marital status of respondent	0.963	0.977
	Expect paid caregiver to be primary caretaker if develop AD	1.277*	1.291*
	Believe an effective AD treatment is available now or will be in five years	1.064	1.051
Demographic controls			
	Age of respondent		
	30 to 49	1.019	1.019
	50 to 64	1.573**	1.582**
	65 to 74	1.702**	1.733**
	75 to 85+	1.465	1.489
	Female	1.18	1.178
	Educational Attainment		
	Middle education	1.112	1.105
	High education	0.846	0.844
	Country of Residence		
	Germany	0.838	0.841
	Poland	1.568**	1.559*
	Spain	1.463*	1.463*
	United States	1.208	1.206
Measure of Psychological Status			
	Would see doctor if showing symptoms of AD	1.44	1.416

AOR = Adjusted odds ratio; reference groups, in order of the categories displayed in the table are: Ages 18 to 29; Low Education; France; * *P *< 0.05,** *P *< 0.01, *** *P *< 0.001.

Measures of perceived risk were the strongest predictors of interest in testing. For example, holding all else constant, the odds of being "very likely" to pursue testing were 76% higher for those worried about developing AD than for those not worried. Expecting to rely on a paid caregiver, older age, and country of residence also remained positive, statistically significant predictors of being interested in early medical testing.

On its own, knowing the disease is fatal had no statistically significant effect on interest in testing (OR = 1.112, *P *>0.05). However, among those who knew the disease is fatal, the predicted probability of expressing interest in testing was higher for those who have a blood relative with AD as opposed to those without (38.7% versus 26.8%). Moreover, although poor health status was predictive of interest in testing in bivariate analyses, it was the worried well who expressed higher levels of interest in our final model. Among those who were worried about AD, those in better health were more likely than those in worse health to desire testing (predicted probabilities of 36.3% versus 31.9%).

These results varied little when we broke them down by country (Table 3). Caretakers, especially those in Poland, report higher interest in testing as opposed to those who have not served as a caretaker or decision-maker for an AD patient (OR = 2.717, *P *< 0.001). Despite different social support systems, interest was also high in most countries among those who expected to rely on a paid caregiver. However, although marital status was not a significant predictor of testing in the aggregate, married respondents in the United States were less likely than single respondents to report a desire for the test (OR = 0.641, *P *< 0.05).

**Table 3:** Country-level logistic regression results evaluating variables predictive of being "very likely" to obtain test

Variable		Entire sample	France	Germany	Poland	Spain	United States
Measures of perceived threat							
	Have/had immediate blood relative with AD	1.312* (0.165)	1.194 (0.336)	1.042 (0.340)	1.739 (0.624)	1.386 (0.389)	1.411 (0.325)
	Respondent is or was decision-maker or caretaker for AD patient	1.306* (0.175)	1.065 (0.362)	0.973 (0.363)	2.717** (0.977)	1.170 (0.307)	1.066 (0.288)
	Worried will get Alzheimer's	1.760*** (0.203)	2.982*** (0.755)	1.647+ (0.469)	1.055 (0.320)	1.594 (0.476)	1.979** (0.432)
	Think AD is fatal	1.112 (0.117)	1.332 (0.302)	1.271 (0.346)	0.758 (0.204)	1.329 (0.316)	0.907 (0.196)
	"Excellent/Good/Very Good" health status	0.972 (0.125)	0.814 (0.261)	0.969 (0.352)	1.028 (0.308)	1.016 (0.306)	0.923 (0.231)
Measures of perceived costs and benefits							
	Marital status of respondent	0.963 (0.113)	1.445 (0.397)	1.383 (0.428)	0.601 (0.191)	1.393 (0.382)	0.641* (0.142)
	Expect paid caregiver to be primary caretaker if develop AD	1.277* (0.146)	0.936 (0.228)	1.991* (0.561)	1.893+ (0.650)	1.359 (0.327)	1.001 (0.224)
	Believe an effective AD treatment is available now or will be in five years	1.064 (0.118)	1.090 (0.276)	0.920 (0.251)	1.196 (0.339)	1.057 (0.242)	1.173 (0.277)
Age of respondent							
	30 to 49	1.019 (0.165)	1.558 (0.578)	2.491+ (1.370)	1.476 (0.595)	0.544+ (0.189)	0.677 (0.221)
	50 to 64	1.573** (0.270)	1.291 (0.563)	3.494* (2.077)	3.649** (1.529)	0.827 (0.329)	1.291 (0.417)
	65 to 74	1.702** (0.345)	2.670* (1.241)	2.762 (1.887)	2.865* (1.411)	0.966 (0.443)	1.322 (0.510)
	75 to 85+	1.465 (0.352)	3.191* (1.757)	2.704 (1.741)	1.452 (0.969)	0.584 (0.395)	1.237 (0.518)
Gender							
	Female	1.180 (0.123)	1.035 (0.241)	0.944 (0.261)	1.342 (0.358)	1.090 (0.245)	1.247 (0.268)
Educational Attainment							
	Middle education	1.112 (0.144)	0.882 (0.270)	0.800 (0.264)	1.491 (0.460)	1.476 (0.412)	0.902 (0.238)
	High education	0.846 (0.109)	0.853 (0.261)	0.576+ (0.182)	0.813 (0.267)	0.976 (0.279)	0.943 (0.236)
Country of Residence							
	Germany	0.838 (0.147)					
	Poland	1.568** (0.271)					
	Spain	1.463* (0.235)					
	United States	1.208 (0.192)					
Measure of Psychological Status							
	Would see doctor if showing symptoms of AD	1.440 (0.341)	0.903 (0.407)	1.055 (0.569)	1.695 (0.844)	3.711+ (2.857)	1.398 (0.631)
Observations		2357	494	423	419	436	585
Wald test		110.47	33.44	23.48	50.6	21.13	34.21
*P*-value		0	0.007	0.102	0	0.174	0.005

+*P *< 0.10,* *P *< 0.05,** *P *< 0.01, *** *P *< 0.001. Table displays adjusted odds ratios and standard errors in parentheses. Country-level sample sizes are reduced due to missing data. Reference groups, in order of the categories displayed in the table are: Ages 18 to 29; Low Education; France

Table 4 shows that in the United States, race and ethnicity were also significant predictors of interest in AD testing. On average, blacks and Hispanics were more likely than whites to report an interest in testing. The predicted probability of pursuing an early medical test for AD for whites was 23.1%, but it was nearly double that for blacks (41.3%) and 35.3% for Hispanics. There were no significant differences between minority groups on rates of interest.

**Table 4:** Logistic regression results evaluating interest in preclinical AD test by race in the United States

Variable		US	Predicted probability
Race			
	White	1.000 (--)	23.1%
	African American	2.391** (0.689)	41.8%
	Hispanic	1.820* (0.499)	35.4%
	Other (Asian, Native American, or Other)	2.259 (1.297)	40.5%
Observations		585	

+*P *< 0.10,* *P *< 0.05,** *P *< 0.01, *** *P *< 0.001. Table displays adjusted odds ratios and standard errors in parentheses. White is the reference category. Sample size is reduced due to missing data. Model controls for measures of perceived threat, perceived costs and benefits, demographic controls, and measure of psychological status.

Lastly, we explored factors associated with being "not at all likely" to pursue early medical testing. Not surprisingly, having a positive information-seeking style, for example, respondent reporting that s/he would visit a physician if exhibiting symptoms of Alzheimer's, was inversely related to being "not at all" interested in testing (OR = 0.534, *P *< 0.01). This suggests that those who would avoid physician visits were also more likely to avoid early medical testing.

## Discussion

This is the first large, international, randomized survey of public interest in potential early medical testing for AD. We find that two out of three respondents would be interested in obtaining a hypothetical early medical test for AD. These rates are similar to results found in a 2011 internet survey conducted in the United States finding high levels of interest in and willingness to pay for AD testing (between 70% and 74.8% of respondents) [29]. In a study of first-degree relatives who had undergone APOE susceptibility testing, fear of developing the disease and a desire for information were among the chief predictors of willingness-to-pay [30].

These results suggest that demand will be highest among those who perceive themselves to be at risk for the disease, including those with a family history of AD, those worried about getting the disease, and those who serve as caregivers or decision-makers for AD patients [17,19,25,26]. Our study adds to the literature by uniquely showing the interactive nature of these measures. While in our bivariate analyses, those in worse health were more likely than healthy populations to desire testing, it was the worried well who expressed higher levels of interest in a hypothetical predictive test, controlling for demographic and social factors. While our own study does not directly ask about motivation for test-taking, studies of other late-onset disorders find that those with high levels of perceived risk view testing as a way of practically and emotionally coping with their worry, gaining control and getting clarity about their future [15,31]. Even in circumstances where no treatment options were available, study participants highlighted the non-medical benefits to test information, highlighting the "value of knowing" and the opportunity to change behaviors, such as getting follow-up care, spending time with family, and arranging their personal finances [16,29].

Previous work on these survey data found that awareness of the disease's fatality was limited, ranging from 33% in Germany to 61% in the United States; however, awareness was higher among blood relatives of AD patients in France, Poland and Spain [23]. We hypothesized that interest in testing would be lower among those who knew the disease was fatal, and even more so among those who genetically had higher chances of developing the disease. Unexpectedly, this knowledge had no significant effect on interest, except, inversely so, among respondents with a blood relative with AD. As noted previously, these respondents may value information over uncertainty, even in the context of potentially negative results [29].

Nevertheless, it could be that rates of interest do not translate into rates of take-up once, and if, a definitive, predictive test becomes available in clinical settings [27]. Yet, we anticipate that high prevlance rates for AD, coupled with its later age of onset and broader media presence, could bolster participation rates beyond those documented for other uncurable, untreatable conditions [27,28].

Middle- to older-age populations who are both closer to the age of onset and more likely to serve as caretakers are also more likely to express interest in testing [28]. Looking forward, as global populations age and as more people gain experience with the disease, the demand for early medical AD testing could rise. We anticipate that this demand will vary by country, with potentially high levels of demand in Poland, where support systems for AD patients are more fragmented and thus planning for care falls to individuals, and Spain, where AD related media has been prevalent and informal care giving is more common [32]. Compounding these factors, we expect interest and utilization of early medical testing for AD across countries to be affected by variations in clinical practice, care resources, cultural norms, disease epidemiology, levels of disease awareness and public policy responses [33,34].

Lastly, compared to whites, blacks and Hispanics living in the United States expressed significantly more interest in predictive testing for AD. This was unexpected given that earlier studies of genetic testing for cystic fibrosis, hereditary breast and ovarian cancers, and Alzheimer's disease, found either no differences across racial groups or that whites were more likely to express interest in genetic testing, seek out genetic counseling, and undergo testing once available [18,35-38]. However, one national telephone survey conducted in 2000 found that African-American and Latino adults, in comparison to whites, were more likely to express interest in genetic testing for untreatable conditions [39].

Previous surveys have found that African American and Hispanic populations had higher levels of treatment optimism, believing scientists were close to finding a cure and that a cure would be available during the study participant's lifetime; moreover, minorities were also more likely than whites to report lower levels of concern about developing AD, which could explain differences observed in our study [35,40,41]. However, we found no statistically significant differences across racial groups on our survey measures of treatment optimism or levels of perceived risk. More work is needed to understand how these beliefs and interest vary across racial groups, and the extent to which individual- and structural-level factors affect rates of take-up once and if testing becomes available [39,42,43].

### Transforming medical and legal landscapes

If an early diagnostic test is indeed developed and demand matches global interest, millions of people in each of the studied countries will become members of a new population and political advocacy group: asymptomatic adults living with a diagnosis of Alzheimer's disease. Consequently, policymakers and clinicians should expect significant changes in the utilization of medical and economic resources and address potential legal obstacles.

In the medical realm, diagnosed but asymptomatic individuals are likely to press for follow-up testing, ongoing medical monitoring, and medical management of potential complications associated with Alzheimer's disease [44]. Given the uncertainty around the disease's pathogenesis and treatment mechanisms, the clinical value of such tests is unclear, and these additional costs could strain already-overburdened health systems, making the tradeoffs involved in allocating medical resources even more difficult [32,45]. However, the non-clinical benefits of testing, such as signing advanced directives and spending more time with family and friends, do provide value and should be appropriately considered in cost-benefit calculations [46].

In the legal realm, early medical testing for AD raises challenging questions related to testing protocols, disclosure practices, confidentiality protections, employment and insurance discrimination, and the availability of follow-up care [47,48]. If not appropriately addressed, any of these issues could pose real barriers to test participation. For instance, insurers, including health, life, disability or long-term care insurers, may want access to private health information to protect against adverse selection. These desires are not unreasonable. In the United States, one study found that individuals who underwent genetic testing for Alzheimer's disease were five times more likely than those who were untested to change their long-term care insurance coverage in the year following testing [49]. Similar results were found among those who were found to be at risk for Huntington's disease [50].

The US and Europe have enacted a range of measures to protect individuals against employment and insurance discrimination on the basis of genetic information, such as the Genetic Information and Nondiscrimination Act of 2008 in the US and the 1999 Oviedo Convention of Human Rights and Biomedicine in Europe. However, research suggests that the laws protecting against genetic discrimination in Europe have had mixed results on providing adequate protections and have not always kept pace with scientific advancements [51].

Furthermore, if an early medical test were developed for Alzheimer's disease that did not involve genetic information, such as blood tests evaluating protein levels, it is unclear to what extent these anti-discrimination protections would apply to diagnosed individuals. Before introducing early medical testing for AD into clinical practice, government leaders will need to examine whether existing protections are sufficient for diagnosed individuals and how these protections affect the viability of voluntary private insurance markets.

### Limitations and opportunities for future work

Future research could build on this analysis in a number of ways. First, imprecision in our survey question may introduce some bias in our results. For example, the phrase "in the future" is used twice: to describe both the potential existence of an early medical test for Alzheimer's, and to refer to the possibility that the respondent will get Alzheimer's disease later in life. Our results encapsulate respondents' beliefs about the timing and availability of such a test, their interest in the actual test, and their level of concern for events that may happen in the future. Although we control for beliefs about scientific advancement, levels of concern about getting Alzheimer's disease, and age in our models predicting interest, future surveys should consider introducing a hypothetical situation in which a test already exists.

Second, we do not include potentially relevant financial, social and emotional variables, such as the respondents' insurance status, willingness to pay for testing, test administration, family size, country-specific policies, AD media coverage, level of religiosity, history of depression or fear of discrimination [30,35,46,52,53]. Third, future surveys should measure whether people would want to take such a test if they were told that no treatment or cure is currently available. Fourth, while we implied that the test would be completely predictive, we did not explicitly state so. As has been done previously, future work could test whether respondents would want to take a test if it were partially or perfectly predictive [25,46]. Lastly, future analyses could enhance this work by including more questions about test motivations, perceived costs and benefits, and measures of psychological style, including the Miller Behavioral Style Scale [22,54,55].

## Conclusions

In summary, our survey indicates that across four European countries and the United States, interest in early medical testing for Alzheimer's disease is high. We expect those with high levels of perceived risk - those who are worried about getting AD as well as those with more experience with the disease, including caregivers and blood relatives of AD patients - will be among those most likely to pursue testing once it becomes available. While early detection could hasten the development of treatment protocols, high demand for testing and the creation of a large group of asymptomatic adults with an Alzheimer's diagnosis could have significant political, economic and legal implications, and could transform the way AD is addressed by countries in the future.

## Abbreviations

AD: Alzheimer's disease; ApoE: apolipoprotein E; HBM: Health Belief Model.

## Competing interests

The authors declare that they have no competing interests.

## Authors' contributions

Each author contributed to the drafting of the manuscript and the interpretation of the data. EW conducted the analysis and drafted initial versions of this article. RB and JB made critical revisions in the article and took the lead in writing the survey used in this analysis. All authors approve of this published draft.
